# Isolation and purification of Cu-free methanobactin from *Methylosinus trichosporium *OB3b

**DOI:** 10.1186/1467-4866-12-2

**Published:** 2011-02-07

**Authors:** Marie-Laure Pesch, Iso Christl, Kurt Barmettler, Stephan M Kraemer, Ruben Kretzschmar

**Affiliations:** 1 Institute of Biogeochemistry and Pollutant Dynamics, Department of Environmental Sciences, ETH Zurich, CHN, Universitätstrasse 16, 8092 Zurich, Switzerland; 2 Department of Environmental Geosciences, University of Vienna, Althanstrasse 14, 1090 Vienna, Austria

## Abstract

**Background:**

The isolation of highly pure copper-free methanobactin is a prerequisite for the investigation of the biogeochemical functions of this chalkophore molecule produced by methane oxidizing bacteria. Here, we report a purification method for methanobactin from *Methylosinus trichosporium *OB3b cultures based on reversed-phase HPLC fractionation used in combination with a previously reported resin extraction. HPLC eluent fractions of the resin extracted product were collected and characterized with UV-vis, FT-IR, and C-1s NEXAFS spectroscopy, as well as with elemental analysis and ESI-MS.

**Results:**

The results showed that numerous compounds other than methanobactin were present in the isolate obtained with resin extraction. Molar C/N ratios, mass spectrometry measurements, and UV-vis spectra indicated that methanobactin was only present in one of the HPLC fractions. On a mass basis, methanobactin carbon contributed only 32% to the total organic carbon isolated with resin extraction. Our spectroscopic results implied that besides methanobactin, the organic compounds in the resin extract comprised breakdown products of methanobactin as well as polysaccharide-like substances.

**Conclusion:**

Our results demonstrate that a purification step is indispensable in addition to resin extraction in order to obtain pure methanobactin. The proposed HPLC purification procedure is suitable for semi-preparative work and provides copper-free methanobactin.

## Introduction

Aerobic methanotrophic microorganisms are Gram-negative bacteria that have the unique ability to use methane as their only source of carbon and energy [1]. In a first step of their metabolic pathway, methanotrophs oxidize methane to methanol, a reaction catalyzed by methane monooxygenase (MMO). Whereas most aerobic methanotrophs produce a membrane-bound, or particulate MMO (pMMO), some strains also produce a cytoplasmic, or soluble MMO (sMMO) under copper-limiting conditions [1,2]. Both types of MMO contain metal atoms which are involved in the electron transfer to the substrate. pMMO contains several copper atoms [3,4] and possibly a diiron center [3], whereas sMMO holds a diiron active site [5,6]. Copper has been found to play a key role in the regulation of pMMO and sMMO expression as well as in the regulation of other metabolic enzymes of methanotrophs [4,5,7,8]. Furthermore, methane oxidation by pMMO critically depends on copper availability [9]. Due to their high copper requirement, methanotrophs necessitate an efficient copper mobilization and uptake system.

Studies conducted in the 1990s provided the first evidence for the production of copper binding compounds (CBC) or copper binding ligands (CBL) released by methanotrophic bacteria under copper-depleted conditions [10-13]. Such copper binding ligands are termed chalkophores, in analogy to siderophores involved in the iron uptake in many organisms. Only recently, researchers have succeeded in isolating and elucidating the structure of a chalkophore from the methanotroph *Methylosinus trichosporium *OB3b, a small chromopeptide showing high affinity for copper that was named methanobactin [14-16].

**Figure 1:**
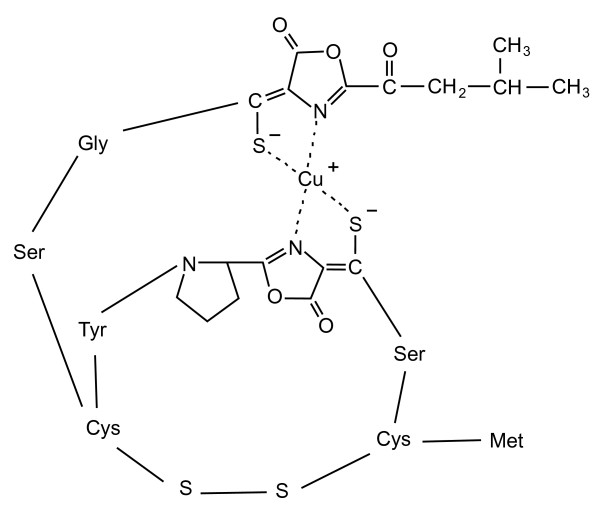
**Structure of methanobactin**. Schematic drawing of copper-bound methanobactin modified from Behling et al. [16].

Copper-bound methanobactin isolated from *Methylosinus trichosporium *OB3b has a molecular mass of 1215 Da and is composed of seven amino acids and two chromophoric residues involved in copper binding [14]. Upon binding to methanobactin, Cu(II) is immediately reduced to Cu(I) [17,18]. Initially, Kim et al. [14] suggested that Cu(I) coordination is associated with two hydroxyimidazolates, each contributing with one N and one S atom to copper binding. Behling et al. [16], however, proposed a revised structure of the oligopeptide methanobactin containing oxazolone rings instead of hydroxyimidazolate rings responsible for copper binding. The structure of the copper-bound methanobactin is shown in Figure 1.

The copper binding peptide methanobactin is not only suspected to be involved in copper acquisition from the extracellular phase [10,17], but it may also function as copper chaperone for pMMO [8,10,19], be responsible for the electron flow to pMMO [19-21], and regulate pMMO expression [10,20]. Furthermore, in vitro studies indicated that methanobactin can act as an oxygen radical scavenger [19,20]. Recent studies revealed the influence of methanobactin in natural systems by effectively mobilizing copper from mineral sources and soils [22]. Additionally, methanobactin was found to promote dissolution of copper-doped silicate [23].

Copper-bound methanobactin has been successfully isolated and purified for characterization and structural elucidation by a two-step purification method [14-16,18]. According to the protocols of the two-step purification method, methanobactin is stabilized with copper and isolated by a solid phase extraction followed by a reversed-phase high-performance liquid chromatography (HPLC) purification step.

Methanobactin obtained by these procedures is suitable for characterization purposes. Sufficient amounts of copper-free methanobactin, devoid of buffers such as phosphate, are prerequisite to further investigations of the biogeochemical functions of chalkophores. Choi et al. [21] described a one-step purification method to gain copper-free methanobactin. The one-step purification method consists of a resin extraction and has been applied in several studies [22-24].

From the analysis of exudates released by *Methylosinus trichosporium *OB3b and isolated following the one-step purification protocol published by Choi et al. [21], however, we found that the elemental composition (mainly C, N, and S) diverges largely from the theoretical composition of methanobactin according to the structure proposed by Behling et al. [16].

Here, we report an improved method yielding highly pure, copper-free methanobactin extending the published one-step resin extraction method with a subsequent purification step with HPLC using a reversed-phase C18 column. Different fractions were collected from HPLC effluent and analyzed in order to separate pure methanobactin and to characterize other compounds that were present in the product of the one-step resin extraction method. Characterization methods included various spectroscopic techniques, such as UV-vis, C-1s near-edge X-ray absorption fine structure (NEXAFS), and Fourier transform infrared (FT-IR) spectroscopy, as well as electrospray ionization mass spectrometry (ESI-MS).

## Materials and methods

### Materials

All reagents used were at least analytical or liquid chromatography grade and purchased from Fluka, Merck, or Sigma-Aldrich. All solutions were prepared with high-purity deionized water (MilliQ, Millipore, ≥18.2 MΩ cm). Glassware was washed in 1 M NaOH and 1 M HCl for 24 hours and rinsed with deionized water. Either amber glassware was used or glassware was wrapped in aluminum foil to exclude light.

### Methanobactin production and purification

*Methylosinus trichosporium *OB3b cells were cultured in nitrate minimal salts medium (NMS) amended with 0.2 μM CuCl_2 _using a BIOSTAT^® ^A plus bioreactor system (Sartorius) as previously described [15,21]. The cultures were grown in batch mode at 30°C and continuously purged with a mixture of air and methane in a ratio of 2:1 (v/v) at a flow rate of 60 mL min^-1^. When the cultures reached an optical density of 0.8-1 at a wavelength of 600 nm, 90% of the culture was harvested and replaced by fresh medium. The harvested medium was centrifuged twice at 9000 *g *for 30 min and immediately vacuum-filtered through a 0.2 μm PTFE filter (Millipore) combined with a glass microfiber pre-filter (Whatman^®^) to remove the cells. The filtrate was then loaded onto a 4 × 30 cm Diaion HP-20 column (Supelco). Subsequently, the column was washed with two column volumes of deionized water prior to elution with 60% methanol. The eluate was freeze-dried immediately.

After this resin extraction, the obtained isolates were dissolved in deionized water in amber glass vials and analyzed by reversed-phase chromatography on a HPLC system (Agilent 1100 Series) with a diode array UV-vis detector. To analyze the different components of the isolates, chromatography was first performed on an analytical ProntoSil 120-5-C18aq column (4 × 250 mm, 5 μm, BISCHOFF Chromatography) with a K2 (4.0 × 20 mm, 5 μm) precolumn. Fractions were then separated using a semi-preparative ProntoSil 120-5-C18aq column (10 × 250 mm, 5 μm, BISCHOFF Chromatography) and a ProntoSil pre-column (8 × 33 mm, 5 μm) in series at a flow rate of 4.4 mL min^-1^, with 10 mM NaCl (solvent A) and methanol (solvent B) as mobile phases. Prior to injection, the column was equilibrated with 40% solvent B and a linear gradient consisting of an initial solvent B concentration of 40% to 50% at 10 min and 100% at 15 min was used. UV-vis absorption of the eluate was monitored at wavelengths of 220, 254, 280, and 390 nm, which is characteristic for methanobactin. Additionally, the absorption spectra ranging from 220 to 600 nm were recorded for each peak. Eluate fractions were collected and freeze-dried for further characterization. Samples were stored at -20°C in the dark to avoid degradation [15].

### Elemental analysis and molecular mass determination

To determine the molar C/N ratio of the collected fractions as well as of the resin extract isolated from the cultures, total organic carbon (TOC) and total nitrogen (TN) were measured with a TOC and TNb analyzer (DIMATOC^®^2000, DIMATEC Analysentechnik GmbH). Total Cu concentrations were determined by graphite furnace atomic absorption spectrometry (GF-AAS) with Zeeman background correction (GTA 120 AA240Z, Varian).

Mass spectra were obtained with a Waters nanoACQUITY UPLC coupled to a Thermo Exactive Orbitrap mass spectrometer using electrospray ionization (ESI-MS) operated in negative ion mode. Freeze-dried material was diluted in deionized water, followed by addition of CuCl_2_. Sample solutions were injected to a nanoACQUITY UPLC trap column (180 μm × 20 mm, 5 μm) for 30 s with 100% 5 mM formic acid at 15 μL min^-1 ^and an analytical Atlantis dC18 column (300 μm × 150 mm, 3 μm) with 30% 5 mM formic acid and 70% acetonitrile at 5 μL min^-1^.

### UV-vis spectroscopy

UV-vis absorption spectra of the resin extract and of the fractions collected during the following HPLC purification step were recorded with a UV-vis spectrometer (Cary50 Bio, Varian). All samples were diluted with deionized water, which was also used as the blank and measured in a 1.0 cm quartz cell between 220 and 600 nm. TOC concentrations of all samples were measured and the absorption spectra of were normalized to the relative carbon content of each fraction.

### C-1s NEXAFS spectroscopy

C-1s near-edge X-ray absorption fine structure (NEXAFS) spectra were collected using the scanning transmission X-ray microscope (STXM) at beamline X-1A of the National Synchrotron Light Source (NSLS), Upton, NY. Sample solutions were freshly prepared by dissolving freeze-dried material in deionized water. Air-dried specimens were prepared by placing a 2 μL droplet of each solution onto an X-ray transparent Si_3_N_4 _window (Silson Ltd., Northampton, UK). For each dry film specimen, spectra were recorded at 15 different spots from 280 to 310 eV in steps of 0.1 eV. The recorded absorbance spectra were background corrected and normalized to the absorbance at 310 eV according to Christl and Kretzschmar [25].

### Fourier transform infrared (FT-IR) spectroscopy

FT-IR absorbance spectra were recorded on a Perkin Elmer Spectrum One FT-IR spectrophotometer equipped with a MIR TGS detector. The spectra were collected in transmission mode in the range of 4000-450 cm^-1 ^with a spectral resolution of 4 cm^-1^. For the analysis, 0.2-0.5 mg of freeze-dried sample material were mixed with 300 mg dried KBr, homogenized with pestle and mortar, and pressed into a pellet under vacuum and under a pressure of 7.5·10^5 ^kPa for 3 min. A background spectrum was recorded with a pellet containing 300 mg KBr. Sample pellets were freshly prepared prior to recording of the spectra. The recorded spectra were background corrected and a linear baseline was subtracted using MATLAB 7.8.0.

## Results and Discussion

### Reserved-phase HPLC analysis

**Figure 2:**
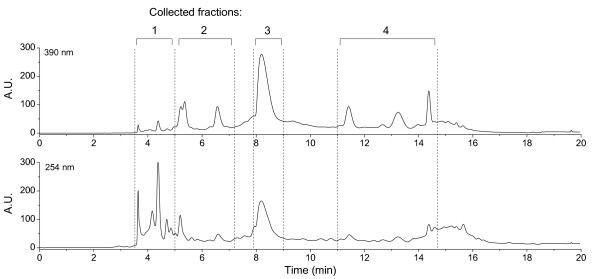
**HPLC elution spectra**. Reserved-phase chromatography of the resin extract isolated from *Methylosinus trichosporium *OB3b cultures was performed with a 10 mM NaCl/Methanol gradient. Absorbance was monitored at 390 nm (top) and 254 nm (bottom).

The product of the previously published resin extraction method [21] was first analyzed with a reversed-phase C18 HPLC procedure. The resin extraction was ultimately developed for routine purification of preparative amounts of high-purity copper-free methanobactin. Here, we used the chromatographic separation to analyze the product obtained from the resin extraction for possible impurities. The chromatogram revealed several peaks at a detection wavelength of 390 nm, with one major peak eluted after 8 min (Figure 2). Detection at a lower wavelength of 254 nm, however, displayed intense signals of fractions eluted with the void volume of the column. The detection of multiple peaks indicates the presence of a number of substances other than methanobactin in the isolate obtained from the cultures. Consequently, the one-step resin extraction procedure is insufficient to obtain pure methanobactin. Four fractions, marked as 1, 2, 3, and 4 in Figure 2, were collected for further characterization using the semi-preparative column with a maximal load of 0.3 mg of the isolate per injection. Fraction 1 mainly consisted of substances with higher absorbance at 254 nm, but lower absorbance at higher wavelengths, whereas fractions 2 and 4 are composed of substances with higher absorbance at 390 nm. Fraction 3 comprises only the peak exhibiting the most intense absorbance at 390 nm.

### C/N ratio

The molar carbon to nitrogen (C/N) ratios of the four HPLC fractions and of the resin extract are shown in Table 1. The theoretical C/N ratio of methanobactin is 4.5 according to the molecular structure proposed by Behling et al. [16] (Figure 1). The mean C/N ratio of the product isolated from 10 separate cultivation and extraction cycles was 7.9 ± 2.4. Total carbon measurements (not shown) revealed that the carbon content was approximately equal between the isolates from different harvests. Nitrogen content, however, was always much lower than the concentrations expected from the molecular structure of methanobactin and varied considerably between the isolates. Thus, a substantial amount of organic impurities are present in the isolates obtained with a single resin extraction.

**Table 1:** Molar C/N ratios.

	C/N	Relative C content	Relative N content
Resin extract	7.9 ± 2.4	100%	100%
Fraction 1	8.7 ± 1.5	35%	23%
Fraction 2	6.3 ± 0.2	13%	14%
Fraction 3	4.7 ± 0.1	32%	42%
Fraction 4	6.1 ± 0.3	20%	21%
Molecular structure of methanobactin	4.5		

Molar C/N ratios of the product isolated from *Methylosinus trichosporium *OB3b cultures with resin extraction and of the four fractions collected during the following HPLC fractionation procedure as well as the molar C/N ratio of methanobactin according to the molecular structure proposed by Behling et al. [16]. Additionally, the relative C and N contribution of fractions 1-4 to the total content of the resin extract is shown.

The C/N ratios of the four fractions shown in Table 1 represent triplicate measurements. Fraction 1 showed the highest C concentrations with a C/N ratio of 8.7, followed by fraction 2 and 4 with C/N ratios of 6.3 and 6.1, respectively. All three fractions exhibited higher C carbon content as expected for methanobactin. Only the C/N ratio of fraction 3 approximately corresponded to the C/N ratio according to structure of methanobactin.

Mass calculations showed that fraction 3 contributed only to about one third of the total carbon content of the product isolated with resin extraction (Table 1). Thus, most of the organic carbon of the product of the one-step resin extraction procedure did not originate from methanobactin.

Since reversed-phase HPLC analysis and C/N ratios revealed the presence of numerous substances other than methanobactin, the composition of the four fractions collection during the second purification step was further characterized by means of spectroscopic techniques.

### UV-vis

**Figure 3:**
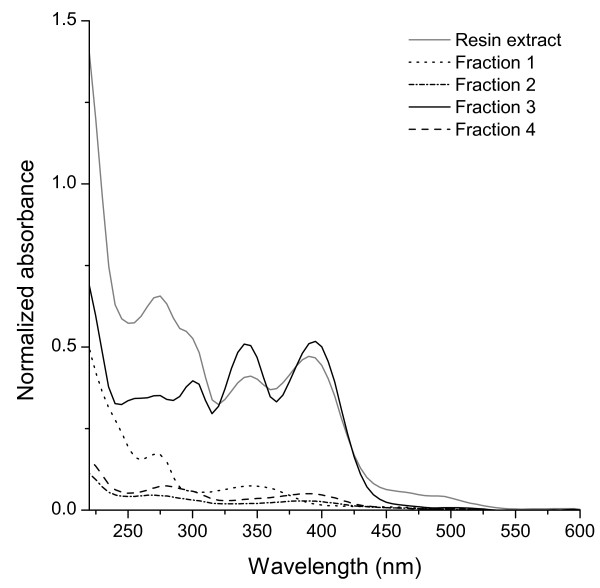
**UV-vis spectra**. UV-vis spectra of the isolate obtained with resin extraction and of fractions 1-4 collected during HPLC fractionation (see Figure 2). Absorbances were normalized to the relative carbon content of each fraction.

UV-vis absorption spectra of the four fractions, as well as of the isolate obtained from the cultures, are depicted in Figure 3. The product obtained from the resin extract showed absorption maxima at 275, 342, and 392 nm and a shoulder at 298 nm. The spectra were similar to the UV-vis spectra of copper-free methanobactin as previously published [15,17]. The absorption maxima at 342 and 392 nm may be assigned to the two chromophoric functional groups of methanobactin involved in Cu binding [15]. According to the revised structure of methanobactin postulated by Behling et al. [16], Cu binding is associated with two alkylidene oxazolone rings, whereof one has a longer conjugated system leading to an absorption maximum at higher wavelengths [17]. Fractions 1-4 showed clearly different spectroscopic properties. Similar to the isolate obtained after the first purification step, fraction 3 exhibited characteristic major peaks at 275, 342 and 392 nm, but the shoulder at 298 nm developed into a peak. Higher intensities of the two peaks at 342 and 392 nm compared to the absorption in the 245-315 nm region were observed for fraction 3. The absorption spectrum of fraction 4 was similar as shown for metal-free methanobactin after methanolysis due to the loss of one oxazolone group [16], leading to the conclusion that fraction 4 is composed of breakdown products of methanobactin and possibly precursor molecules from lysed cells. Analogously, fractions 1 and 2 may consist of breakdown products or by-product produced by *Methylosinus trichosporium *OB3b.

Therefore, UV-vis spectroscopy suggested that methanobactin was eluted with fraction 3. Due to their low absorbance in the range above 300 nm, fractions 1, 2 and 4 contributed only marginally to the absorption spectra of the isolate obtained with resin extraction, even though these fractions contain two third of its organic carbon. Thus, UV-vis spectroscopy is not a suitable tool to detect impurities in partially purified methanobactin.

### C-1s NEXAFS

**Figure 4:**
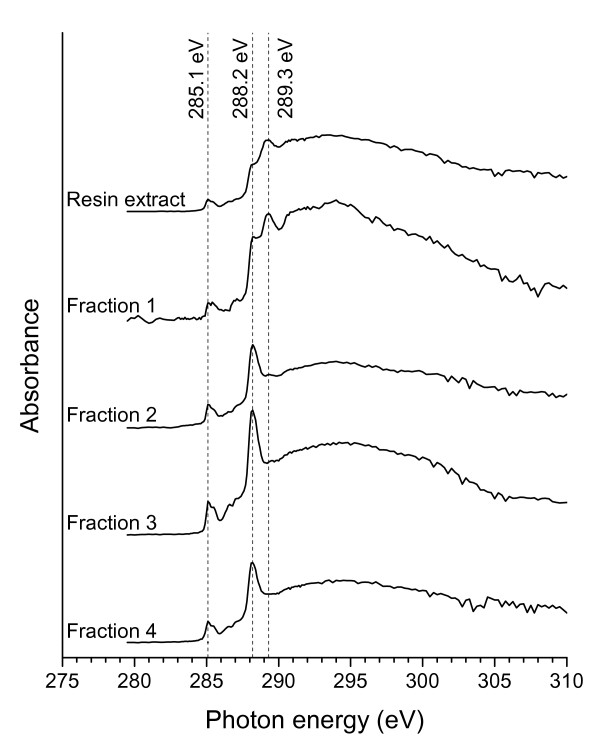
**C-1s NEXAFS spectra**. C-1s NEXAFS spectra of the isolate obtained with resin extraction and of fractions 1-4 collected during HPLC fractionation (see Figure 2). All spectra represent averages of 15 measurements collected on different spots on dry film specimens.

The C-1s NEXAFS spectra of the product isolated from *Methylosinus trichosporium *OB3b cultures and of the fractions collected with HPLC are shown in Figure 4. The spectrum of fraction 1 differed clearly from the spectra of fractions 2-4. Fraction 1 showed an intense peak at 289.3 eV, but lower resonance at 288.2 eV. In contrast, fraction 3 revealed an intense absorbance at 288.2 eV. Furthermore, the spectrum of fraction 3 showed peaks at 285.1 eV, 285.4 eV, and 287.0 eV as well as at 286.6 eV and 287.7 eV. Fractions 2 and 4 showed similar spectroscopic properties as did fraction 3.

Resonances ranging from 288.6 to around 289 eV have been previously reported for the C-1s → π*_C=O _transition of carboxyl carbon of amino acids [26-29]. The position of the carboxyl peak may be lowered by an energy shift of up to 0.4 eV towards 288.2 eV due to the formation of peptide bonds [26,30]. Therefore, the intense signal at 288.2 eV indicates the presence of peptides in fraction 3. Peaks at 285.1 eV and 285.4 eV may be assigned to π*_C=C _states of aromatic carbon connected to C or H [26,27,29], whereas the peak at 287.0 eV may be attributed to C-1s → π*_C=C _transition of aromatic carbon bound to an O atom [28]. Thus, all three peaks can be ascribed to the tyrosine group of methanobactin. The peak at 287.0 eV may also result from π* transitions of C=N bonds [26] present in the two oxazolone rings of methanobactin. The absorption band at 286.6 eV may result from C-1s → π*_C=C _transition from ring structure substituted to N of the oxazolone binding group. Kaznacheyev et al. [27] reported similar resonances for imidazol and histidine amino acids groups. The resonance at 287.7 eV may be related to the C-1s → σ*_C-H _transition or to the C-1s → σ*_C-S _transition, thus, indicating the presence of pyrrolidine, cysteine and methionine units. All absorption bands of the C-1s NEXAFS spectrum of fraction 3 can be assigned to functional groups of methanobactin. Comparison of fractions 2 and 4 with fraction 3 showed that these fractions are also peptide-like material. Together with UV spectroscopic results discussed above, this suggests that fractions 2 and 4 contain breakdown products of methanobactin.

The major part of fraction 1, however, is clearly not peptide-like organic material because the C-1s NEXAFS spectrum showed only a weak peak at 288.2 eV. C-OH moieties, indicated by the strong resonance at 289.3 eV [29], may be partly polysaccharide-like compounds, which are not retained by the C18 column used for fractionation of resin extracts. Similar C-1s NEXAFS spectra were reported for L-(1)-arabinose [29] and xanthan [31]. Polysaccharides are known to contribute to a large extent to microbial extracellular polymeric substances [32]. Likewise, methanotrophic bacteria, including *Methylosinus trichosporium *OB3b, are known to produce extracellular polysaccharides [33]. As a consequence, we conclude that fraction 1 contains mainly polysaccharides, e.g. exopolysaccharides released by *Methylosinus trichosporium *OB3b during cultivation or dissolved decomposition products of lysed cell material.

Principle component analysis of C-1s NEXAFS spectra of the product isolated with resin extraction from *Methylosinus trichosporium *OB3b cultures revealed the presence of two main components, namely a polysaccharide-like and a peptide-like component. Estimations with all recorded C-1s NEXAFS spectra indicated that fraction 1 contributed one third to the total carbon content of the product isolated with resin extraction. This finding is in accordance with the organic carbon mass balance as given in Table 1.

### FT-IR spectroscopy

**Figure 5:**
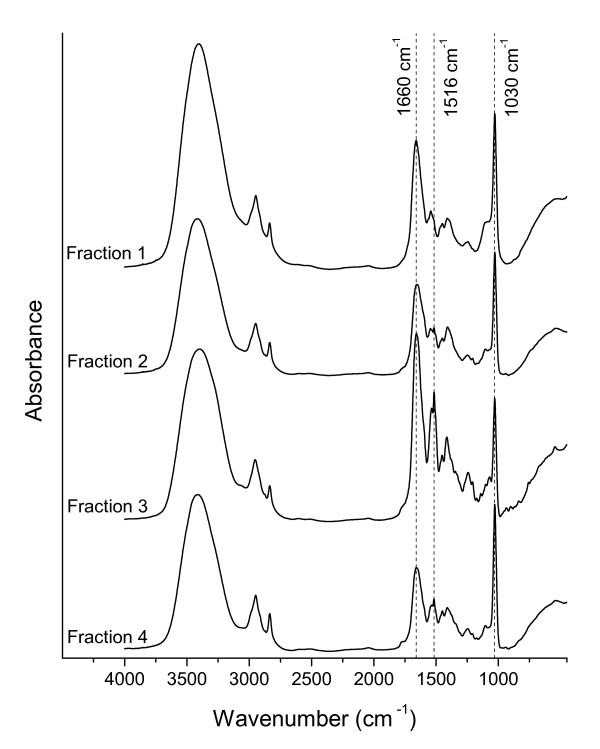
**FT-IR spectra**. FT-IR spectra of fractions 1-4 collected during HPLC fractionation (see Figure 2) of the resin extract. Spectra were recorded in transmission mode from KBr pellets containing 0.2-0.5 mg of freeze-dried sample material.

FT-IR spectra of all fractions collected during the purification of copper-free methanobactin are shown in Figure 5. Peak assignments are based on values published by Bellamy [34] and Parker [35]. A broad intense band at about 3380 cm^-1 ^results from stretch vibrations of H-bonded hydroxyl (OH) groups and N-H stretch of secondary amides. Weaker bands at 2940 and 2840 cm^-1 ^can be attributed to C-H stretching of aliphatic CH_3 _and CH_2_. A weak shoulder at 1760 cm^-1 ^may result from C=O stretching of COOH and ketones. The intense band at 1660 cm^-1 ^can be assigned to C=O stretching of amides (amide I band). Bands at 1540 and 1516 cm^-1 ^can be ascribed to the amide II band of secondary amides found at 1570-1510 cm^-1 ^in solids, which result from a mixed vibration involving N-H bending and C-N stretching [35]. Peaks around 1410 cm^-1 ^can be attributed to O-H deformation, CH_3 _bending, C-O stretching of phenolic OH and COO^- ^antisymmetric stretching. The small band at around 1245 cm^-1 ^can be assigned to C-O stretching and OH deformation of COOH. The intense peak at 1030 cm^-1^, however, results from C-O stretching as present, e.g., in aliphatic esters or polysaccharide-like substances.

The comparison of the different fractions demonstrated that the IR spectra were dominated by absorption bands at 1660, 1540, 1516, and 1030 cm^-1 ^with varying intensities. The intensity ratio calculated for the bands at 1660 cm^-1 ^and 1030 cm^-1 ^revealed a stronger absorption at 1660 cm^-1 ^relative to the absorption at 1030 cm^-1 ^for fraction 3 than for fractions 1, 2, and 4 (Table 2). Similar results were obtained for the intensities of absorption bands at 1540 and 1516 cm^-1 ^relative to the absorption at 1030 cm^-1^. The sum of the absorption bands characteristic for amides (1660, 1540, and 1516 cm^-1^) relative to the absorption at 1030 cm^-1 ^was clearly higher for fraction 3 compared to fractions 1, 2, and 4, thus indicating a more pronounced peptide-like character for fraction 3. Fractions 2 und 4 showed similar absorption properties as did fraction 3 in the region from 1500-1200 cm^-1^, but lower absorption at 1660 cm^-1 ^compared to the absorption at 1030 cm^-1^. Fractions 2 and 4 may therefore consist of degradation products of methanobactin. Likewise, fraction 1, containing mainly polysaccharide-like material according to C-1s NEXAFS spectroscopy (*vide supra*), might also include peptides, originating for example from degradation of lysed cells. Accordingly, FT-IR spectra confirmed the results shown with C-1s NEXAFS spectroscopy. NEXAFS spectra as well as the relative intensities of the FT-IR absorption bands can be used as a reference for future investigations.

**Table 2:** Relative intensities of the FT-IR absorption bands of fractions 1-4 collected during the HPLC fractionation procedure.

	$\frac{1660\;cm^{-1}}{1030\;cm^{-1}}$	$\frac{1540\;cm^{-1}}{1030\;cm^{-1}}$	$\frac{1516\;cm^{-1}}{1030\;cm^{-1}}$	$\frac{(1660+1540+1516)\;cm^{-1}}{1030\;cm^{-1}}$
Fraction 1	0.82	0.36	0.30	1.49
Fraction 2	0.73	0.37	0.37	1.47
Fraction 3	1.53	0.92	1.05	3.50
Fraction 4	0.56	0.30	0.34	1.21

### Purity and stability of isolated methanobactin

**Figure 6:**
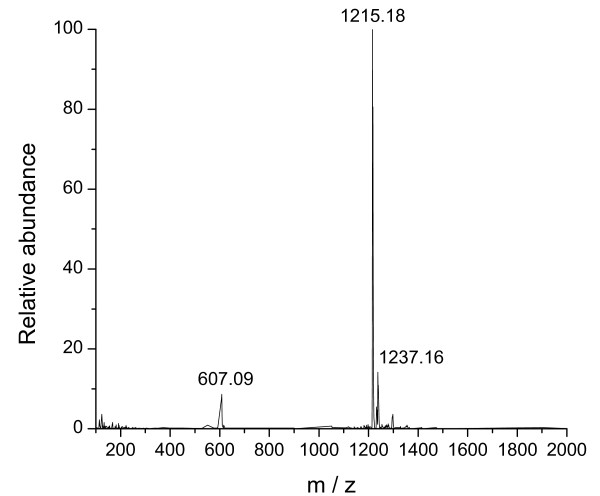
**Negative ion ESI-MS spectrum of copper-bound methanobactin (m/z 1215.18)**.

To further support the conclusion that methanobactin was eluted in fraction 3, this fraction was also analyzed with ESI-MS after copper addition for stabilization. ESI-MS analysis showed an intense signal at m/z 1215 [M - 2H + Cu^+^]¯, thus confirming the presence of methanobactin in fraction 3 (Figure 6). Furthermore, the spectra exhibited a molecular ion at m/z 1237, resulting from Cu-bound methanobactin with one Na atom adduct.

Copper analysis of fraction 3 (conducted with GF-AAS prior to copper addition) revealed a molar copper-to-methanobactin ratio of 0.0027. Methanobactin concentration was calculated from the carbon content of fraction 3. Consequently, methanobactin obtained with the combined resin extraction-HPLC procedure is virtually copper-free. To determine the stability of copper-free methanobactin after purification and storage, freeze-dried samples of fraction 3 were dissolved in deionized water and re-chromatographed using the HPLC procedure which was used for fractionation of resin extracts. Re-injection of copper-free methanobactin showed that a minor contribution of breakdown products was formed. An addition of CuCl_2 _to the very same sample led to a chromatogram with a single peak (for chromatograms see Additional file 1: Figure S1). These results indicate that the small amounts of breakdown products are formed during chromatography and that methanobactin can be stabilized by Cu addition.

## Conclusions

The analysis of resin extracts of harvested *Methylosinus trichosporium *OB3b culture medium revealed that methanobactin was present in the extract, but it contributed only 32% to the total isolated organic carbon of the extract. The extracted organic substances other than methanobactin were found to be peptide compounds as well as more polar compounds exhibiting primarily a polysaccharide-like character. We suggest based on our spectroscopic results that the peptide-like organic impurities represented mainly degradation products of methanobactin, whereas the polysaccharide-like material originated from exopolysaccharides or the decomposition of dead cells. The large variety of organic impurities detected with HPLC as well as the high quantity of these impurities present in the resin extract clearly demonstrated the need for a follow-up purification procedure in order to isolate pure methanobactin appropriate for experimental research on methanobactin. The presented HPLC fractionation using a C18 column was proven suitable for purification. We propose using a two-step procedure consisting of resin extraction followed by HPLC fractionation to isolate and purify methanobactin from *Methylosinus trichosporium *OB3b cultures. This procedure can be used for semi-preparative work. The isolated methanobactin obtained with this two-step procedure is virtually copper-free and thus well-suited for studies investigating copper binding properties of methanobactin as well as the role of methanobactin for copper acquisition of methanotrophs from organic and mineral sources.

## Competing interests

The authors declare that they have no competing interests.

## Authors' contributions

MLP developed the purification method, carried out all analysis, and wrote the manuscript as part of her PhD thesis. IC conducted NEXAFS analysis. KB helped to develop the HPLC purification method. IC, SK, and RK initiated and supervised the study, and were involved in planning experiments, interpreting data, and revising the manuscript for publication. All authors read and approved the final manuscript.

## Supplementary Material

Additional file 1 **Figure S1 Chromatograms of isolated methanobactin**. HPLC elution spectra recorded at 390 nm and 280 nm are shown for isolated copper-free and copper-stabilized methanobactin. Click here for file
