# Supplementary material for: Isolation and purification of Cu-free methanobactin from Methylosinus trichosporium OB3b
Source: Geochem Trans. 2011 Feb 7;12:2. doi: 10.1186/1467-4866-12-2 (PMC3044663; doi:10.1186/1467-4866-12-2)
Supplement: Additional file 1 — Figure S1 Chromatograms of isolated methanobactin. HPLC elution spectra recorded at 390 nm and 280 nm are shown for isolated copper-free and copper-stabilized methanobactin. [file 1467-4866-12-2-S1.PDF]

Additional file 1

Chromatograms of isolated methanobactin

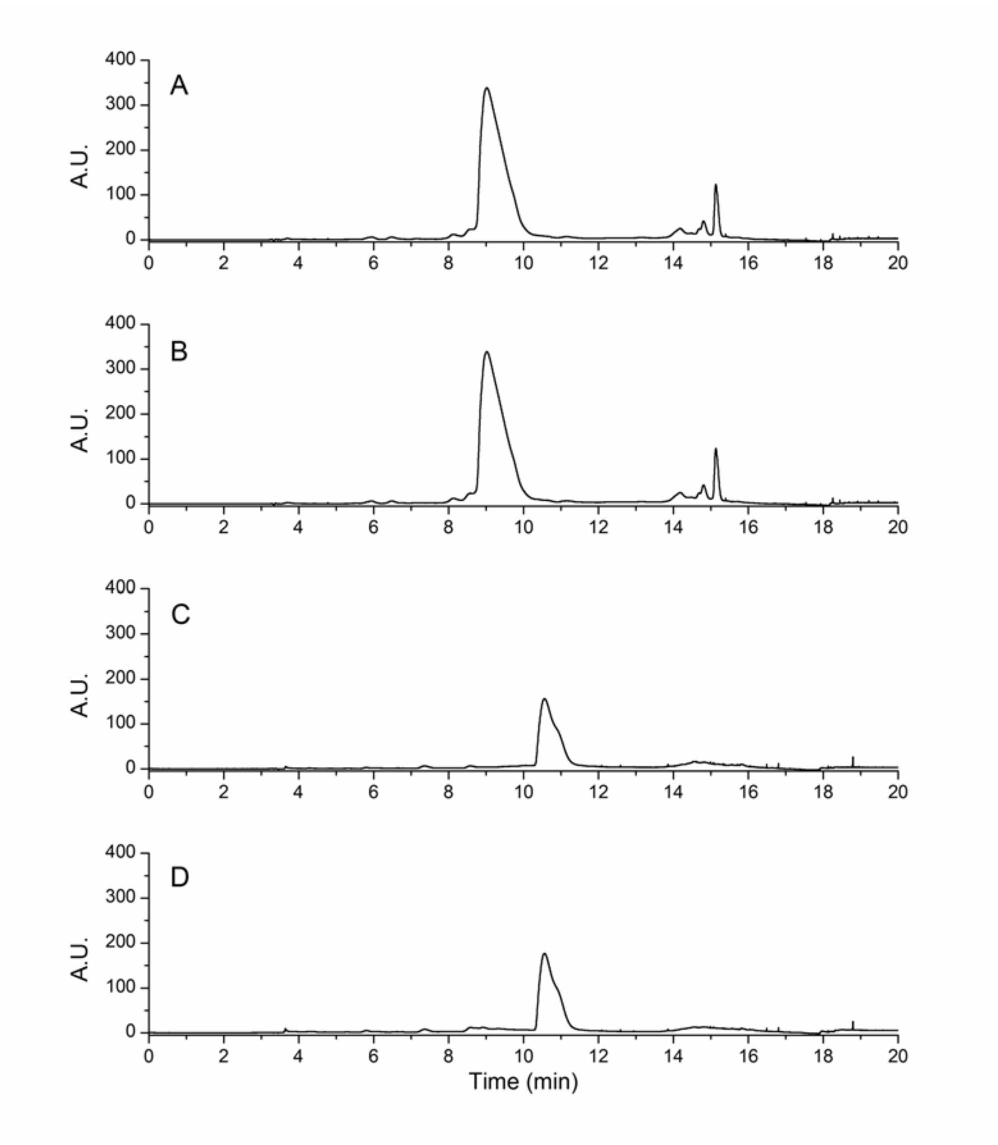

Figure S1 HPLC elution spectra. The stability of isolated methanobactin was assessed with HPLC analysis. Freeze-dried isolates (fraction 3 of resin extracts) were dissolved in deionized water and analyzed using the HPLC procedure which was used for fractionation of resin extracts. Absorbance of eluates is shown for copper-free methanobactin at (A) 390 nm and (B) 280 nm and for methanobactin after stabilization with $CuCl_2$ at (C) 390 nm and (D) 280 nm.
